# The Time Course of Corticospinal Excitability during a Simple Reaction Time Task

**DOI:** 10.1371/journal.pone.0113563

**Published:** 2014-11-18

**Authors:** Michael Kennefick, Dana Maslovat, Anthony N. Carlsen

**Affiliations:** 1 School of Human Kinetics, University of Ottawa, Ottawa, Canada; 2 Department of Kinesiology, Langara College, Vancouver, Canada; 3 School of Kinesiology, University of British Columbia, Vancouver, Canada; University Medical Center Goettingen, Germany

## Abstract

The production of movement in a simple reaction time task can be separated into two time periods: the foreperiod, which is thought to include preparatory processes, and the reaction time interval, which includes initiation processes. To better understand these processes, transcranial magnetic stimulation has been used to probe corticospinal excitability at various time points during response preparation and initiation. Previous research has shown that excitability decreases prior to the “go” stimulus and increases following the “go”; however these two time frames have been examined independently. The purpose of this study was to measure changes in CE during both the foreperiod and reaction time interval in a single experiment, relative to a resting baseline level. Participants performed a button press movement in a simple reaction time task and excitability was measured during rest, the foreperiod, and the reaction time interval. Results indicated that during the foreperiod, excitability levels quickly increased from baseline with the presentation of the warning signal, followed by a period of stable excitability leading up to the “go” signal, and finally a rapid increase in excitability during the reaction time interval. This excitability time course is consistent with neural activation models that describe movement preparation and response initiation.

## Introduction

In order to enable fast responses in simple reaction time (RT) paradigms (where the required motor action is known in advance), cortically-mediated motor preparatory processes are thought to occur during the RT foreperiod, between the warning signal (WS) and the go-signal. Previous studies that have used electroencephalography (EEG) to measure brain activity during self-initiated voluntary movements have shown increases in activity over motor areas that can be detected 1 to 2 s preceding movement onset. This so-called Bereitschaftspotential or “readiness potential” is thought to originate bilaterally from the supplementary motor area and the anterior cingulate, which both project to the corticospinal tract and can directly influence spinal motor networks, as well as the contralateral motor cortex [1]. This readiness potential is typically associated with a corresponding increase in corticospinal excitability (CE), and has been suggested to indicate that the brain is preparing to execute a movement [2]. These studies suggest that an increasing level of neural activation related to the process of motor preparation occurs for simple RT tasks provided there is enough time and information available between the WS and the imperative stimulus (IS). Wickens and colleagues [3] proposed a model in which the activation level of a group of cortical motor neurons related to the performance of a specific action (known as a “cell assembly”) is increased to an initial steady state which is held at a level below the threshold level for motor response production. Following the IS, the eventual triggering (i.e. initiation) of the response results from additional input of activity causing “ignition” of the cell assembly - a spread of neural activity that excites movement-related corticospinal neurons. Therefore, for producing fast responses, an ideal level of motor preparatory activation in the cell assembly would be as close as possible to the ignition point, so that only a minimal amount of additional input would cause the response to be initiated. However, keeping the level of activation near threshold is inherently difficult due to the variability of noise within the system, which can be caused by sensory noise, cellular noise and/or motor noise [4]. Several models describing the preparation and initiation processes occurring during the production of movement have been proposed [5]–[8], which can be directly tested using transcranial magnetic stimulation (TMS) to measure CE.

Many studies have examined CE *following* the IS in simple RT tasks by measuring motor evoked potential (MEP) amplitudes from the prime movers [9]–[12]. MEP amplitudes typically increase anywhere from 100 ms [9], [10] to 50 ms [12] prior to the response onset in the muscle, as measured by electromyography (EMG). These studies indicate there is an increase in CE during the RT interval as the onset of EMG approaches, which is associated with the initiation of the prepared response. Conversely, a comparatively small body of literature exists in which CE was examined *prior* to the IS. For example, Touge and colleagues [13] recorded MEPs during a simple RT task with a short (500 ms) foreperiod. They showed that during the foreperiod there was a decrease in the amplitude of MEPs as the go signal approached, compared to control MEPs (which were recorded in trials in which no WS was presented). It was suggested that subjects likely maintained a high level of preparatory activity throughout the experiment, and that the decrease in CE may have been caused by inhibition within the motor cortex acting to prevent the premature release response just prior to the go-signal [14]. Indeed, using single cell recordings, Prut and Fetz [15] suggested that inhibitory modulations may reflect a general “braking” mechanism in which the tendency to initiate a movement during the foreperiod is suppressed and that the “brake” is released once the IS appears and movement is initiated. In contrast, when Touge and colleagues employed a longer (2 s) foreperiod, MEP amplitudes were no different compared to control, possibly because this time interval is more difficult to estimate, and thus inhibition of premature responses was less likely to be required.

In summary, although the excitability of the corticospinal tract is relatively well defined during the response preparation and initiation phases of a simple RT task, a cohesive picture of the entire time course of corticospinal activation has not been reported in a single study. As such, no direct comparisons of CE before and after the IS are available. In addition, studies examining CE levels during the foreperiod have typically compared activation to the first measured CE value in the foreperiod, or to CE measured in trials without a WS, rather than to a baseline (i.e., resting) value. This comparison may not capture the time course of CE activation relative to a resting level as response-related preparatory activity may have already occurred at the first measurement point. Therefore, the purpose of the current experiment was to characterize motor cortical excitability related to the prime mover at various time points between the WS and movement onset in a simple RT task, in comparison to a true resting baseline value measured during the rest interval between trials. The goal was to provide a description of when and at what rate changes in CE occur during movement preparation and initiation, allowing for a greater understanding of cortical activation related to the processes of preparation and initiation, and how this activation matches with existing models.

## Materials and Methods

### Ethics statement

Fully informed, written consent was obtained from all participants prior to taking part in the study. The study was conducted in accordance with ethical guidelines approved by the University of Ottawa's Research Ethics Board (REB approval: H03-12-03) and conformed to the guidelines of the Declaration of Helsinki.

### Participants

A power analysis [16] performed prior to testing indicated a minimum of fifteen participants were required to reach the conventional power levels. In order to exceed this requirement, eighteen healthy volunteers (9F, 9M; 24±5 years) with normal or corrected to normal vision, and with no history of neurological, sensory, or motor disorders participated in this study. All participants were classified as right-handed or ambidextrous based on the Edinburgh Handedness Inventory [17]. Testing of each participant took place in a single session, and required approximately 1.5 hours to complete.

### Experimental set-up and task

Participants sat comfortably facing a 17 inch LCD computer monitor with their right arm pronated and resting on a flat surface, and completed a simple RT task in which they performed a single button-press movement, in response to a visual IS. This movement required the pressing of a telegraph key (Ameco AM-K4B) by flexing the second digit (index finger) using the flexor digitorum superficialis (FDS) muscle. Participants were instructed to execute this movement as quickly as possible following the IS. The presentation of a visual WS, a blank black outlined box (6 cm×5 cm) located in the center of the screen, indicated the start of the trial, which was followed by a fixed foreperiod (1500 ms). The visual IS consisted of the interior of the box turning green, prompting the participant to initiate their movement. A fixed foreperiod was utilized to ensure that MEP data collected during the foreperiod were time-locked to the same intervals across all participants. Displacement RT feedback was provided on the computer monitor after each trial, determined from the closing of the circuit of the telegraph key switch. Prior to the start of the testing session, each participant performed a practice block consisting of 10 RT trials. The practice trials were identical to the testing trials, with the exception that TMS was not applied.

**Figure 1:**
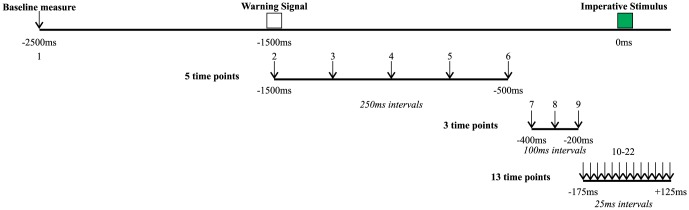
Visual representation of the TMS stimulation points with respect to imperative stimulus onset, represented by downwards pointing arrows.

Following the practice session, participants performed 8 blocks of 24 trials, consisting of 22 RT trials and 2 catch trials per block, for a total of 192 testing trials. TMS stimulation (see below for details) was applied over motor cortex at 22 time points with respect to the IS (each stimulation time point occurred once per testing block). TMS was applied at −2500 ms (during the inter-trial interval, 1 s prior to the WS), 5 equally spaced time points between −1500 ms and −500 ms, 3 time points between −400 ms to −200 ms, and 13 time points between −175 ms to +125 ms (see figure 1 for visual representation). MEPs collected at the first stimulation point (−2500 ms) were considered as baseline since these occurred during the inter-trial interval, prior to the WS and thus it is presumed that the participant was fully at rest. The inter-trial interval varied randomly in duration between 6 s and 8 s to avoid anticipation of trial onsets. Catch trials consisted of the presentation of WS followed by TMS, but without the IS. This ensured that the participant reacted only when he or she was meant to. In order to encourage advance preparation, a points reward structure was provided to the participant based on RT whereby when participants produced a RT below a predetermined criterion, points were awarded. Alternatively, executing a movement during catch trials resulted in a loss of points.

### Transcranial magnetic stimulation

TMS was applied using a figure-8 magnetic coil (70 mm; Magstim 200^2^, Magstim Company Ltd, UK). Prior to testing, the coil was placed over the optimal location for eliciting MEPs from the right flexor digitorum superficialis (FDS) muscle, with the handle of the coil pointing backwards at a 45° angle. The optimal location was found by first finding the midpoint between the nasion and inion, and the left and right preauricular notches. From this midpoint, a location 5 cm lateral and 1 cm posterior was marked on the participant's scalp using a red grease crayon. The optimal location was then found by delivering test pulses at various scalp locations around this mark and determining the location that resulted in consistently large MEPs. Resting motor threshold (RMT) was determined at rest to the nearest 1% of stimulator output using the Rossini-Rothwell [18] method (defined as the minimum intensity required to evoke MEPs above 50 µV in at least 5 out of 10 trials). The magnetic coil was held stationary over the optimal location by the experimenter and the position was maintained by holding the coil in the reference position on the head with the assistance of neuronavigation hardware and software (ANT Neuro Visor 2, Madison, WI). During experimental trials stimulus intensity was adjusted to 110% of RMT as similar intensities have been used previously to probe changes in CE [12], [19].

### Recording equipment

Surface EMG data was collected from the muscle belly of the right FDS muscle using a bipolar preamplified (gain = 10) surface electrode (Delsys Bagnoli DE-2.1) connected via shielded cabling to an external amplifier (Delsys Bagnoli-8). The electrode was placed parallel to the muscle fibres, and attached to the skin using double sided adhesive strips. A grounding electrode (Dermatrode HE-R) was placed on the participant's right lateral epicondyle. The site of each electrode was prepared and cleaned using abrasive skin prepping gel and alcohol wipes to ensure minimal electrical impedance. Unfiltered EMG and telegraph key data was digitally sampled at 1 kHz (National Instruments PCI-6024E) beginning 500 ms prior to the first stimulation point for a total duration of 3500 ms using a custom made program written in LabVIEW (National Instrument Inc.) and stored for offline analysis.

### Data reduction

MEP amplitudes, the time between TMS pulse and EMG onset, and the time of movement onset were calculated. Trials in which no MEPs were observed were rejected (3.8%), typically when the MEP occurred during the EMG burst, as it is inherently difficult to distinguish MEPs from EMG activity associated with the actual movement (65% of the rejected trials occurred for time points following the IS). MEP amplitudes were quantified by calculating the peak-to-peak voltages of the evoked responses, expressed as a percentage of the baseline MEP amplitude. EMG burst onsets were marked by a custom computer algorithm written in LabVIEW which defined EMG onset as the point at which EMG activity reached a value of 2 standard deviations above baseline levels (mean of the first 100 ms of data collection) and remained at that level for more than 20 ms [20]. EMG markers were visually verified and manually adjusted if necessary to compensate for any errors due to the strictness of the algorithm. Movement-related EMG activity in the FDS muscle was marked for each trial. In order to analyze the data relative to EMG onset, the difference in time between the onset of agonist EMG and the presentation of TMS was calculated. Errors in movement (e.g., multiple button-press movements; 3.4%) and trials in which EMG onset occurred in less than 50 ms following the IS (i.e., anticipation; 4.8%) were removed.

### Statistical Analysis

Dependent variables were analyzed using a one-way repeated measures analysis of variance to determine if differences exist between TMS presentation times (e.g. the 22 time points between −2500 ms prior to the IS and +125 ms following the IS). MEP data was normalized to baseline values measured at time point 1 in order to account for the variability in individuals' resting CE levels. Preplanned comparisons using uncorrected t-tests were administered to determine the exact locus of any significant differences in the overall CE time course. Preplanned uncorrected t-tests were used due to the large number of comparisons, and correcting for all these possible comparisons may lead to an increased probability of committing a type II error. Differences with a probability of less than 0.05 were considered significant. Secondary post-hoc analyses are described below.

## Results

### Experimental parameters

Across participants, RMT was 47±10% of maximal stimulator output, mean test stimulus was 52±11% of stimulator output, and mean premotor RT (time from IS to EMG onset in prime mover) was 185 ms±21 ms.

### MEP amplitude

**Figure 2:**
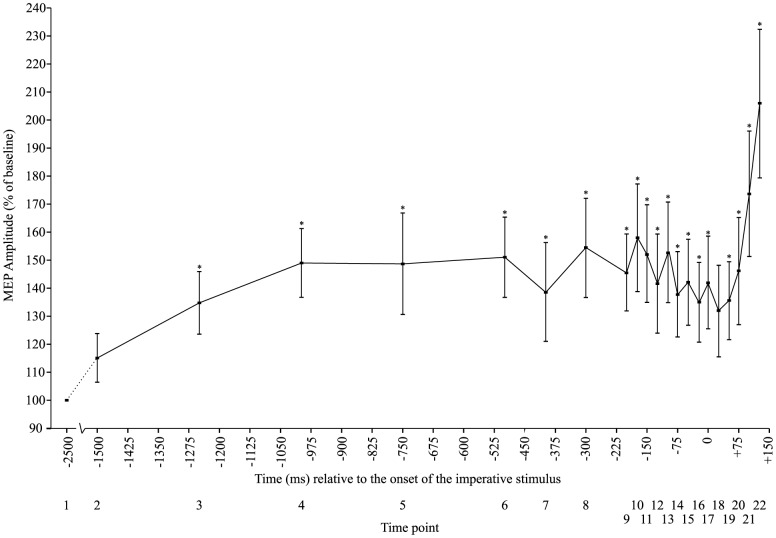
Mean (+/− SE) motor-evoked potential (MEP) amplitude relative to the onset of the imperative stimulus expressed as a percentage of baseline.

At baseline (−2500 ms time point), between-subject mean raw MEP amplitude was 0.23 mV±0.10 mV. Analysis of normalized MEP amplitude revealed a significant main effect of time (*F*(21,357) = 4.560, *p*<0.001, η_p_^2^ = 0.212). T-tests comparing each time point to baseline revealed a significant difference between the baseline (time point 1) and every other time point in the current study, with the exception of time point 2 (−1500 ms) (*p* = 0.1) and time point 18 (+25 ms) (*p* = 0.066). This indicates that there was increased CE, relative to the resting baseline levels, throughout the foreperiod and RT interval (fig. 2).

### MEP time-course breakdown

To protect against making errors associated with a large number of comparisons, MEPs recorded during several different timeframes (or “epochs”) during the RT task were analyzed separately. The first epoch was thought to capture preparatory processes and included the inter-trial baseline MEP, as well as MEPs recorded between −1500 ms to −500 ms with respect to the onset of the IS. The second epoch, which encompasses the stimulation time points from −500 ms until the IS was analyzed in order to be able to compare this time frame to that described in previous studies [13], [21]. Finally a third epoch was analyzed encompassing the stimulation time points from the IS onward until task-related EMG onset to examine initiation-related processes.

#### MEP amplitude during 1st epoch

**Figure 3:**
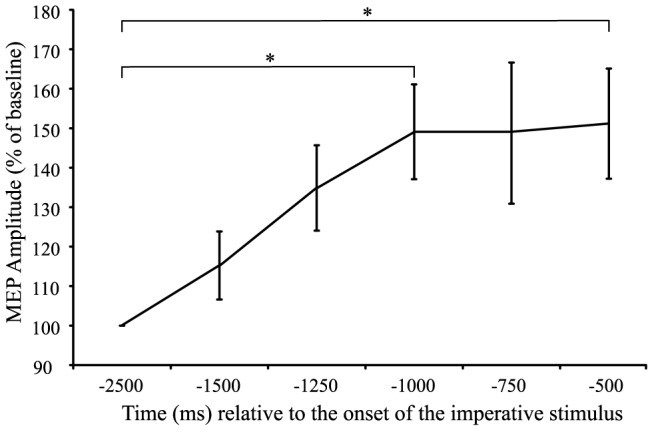
Mean (+/− SE) motor-evoked potential (MEP) amplitude expressed as a percentage of baseline for the time encompassing −2500 ms to −500 ms relative to the onset of the imperative stimulus. Asterisks (*) denote a significant increase in MEP amplitude during this preparation phase compared to baseline.

For a simple RT task with a relatively long foreperiod, it would be expected that response preparation would occur at some time following the WS, with the state of “readiness” maintained until the appearance of the IS. Increases in CE during the foreperiod have previously been shown in the literature, albeit with different reference times and baselines [9]–[12]. In the current experiment a one-way, six factor (Time: −2500 ms, −1500 ms, −1250 ms, −1000 ms, −750 ms, −500 ms) repeated measures ANOVA was undertaken in order to examine any differences in MEP amplitude relative to baseline during the first epoch. The results indicated a significant main effect of time (*F*(5,85) = 5.515, *p*<0.001, η_p_^2^ = 0.245). Post-hoc analyses using Bonferonni corrected student's t-tests were used to determine the locus of the differences. The results indicated a significant difference between MEP amplitude measured at baseline (time point 1: −2500 ms) and time points 4 (−1000 ms), and 6 (−500 ms), confirming that an increase in CE relative to baseline occurred during the early component of the foreperiod (figure 3).

#### MEP amplitude during the 2^nd^ epoch

In order to analyze the second epoch and understand how CE level changed as the IS approached, a one-way, twelve factor (Time: −500 ms, −400 ms, −300 ms, −200 ms, −175 ms, 150 ms, −125 ms, −100 ms, −75 ms, −50 ms, −25 ms, 0 ms) repeated measures ANOVA, using polynomial contrasts adjusted for unequal intervals between time points, was undertaken to examine the 500 ms preceding the IS. This time frame was chosen because several previous studies [13], [21] have examined foreperiod CE during this time frame. The analysis indicated a non-significant main effect of time (*F*(11,187) = 1.015, *p* = 0.434, η_p_^2^ = 0.056), as well as a non-significant trend analysis (linear, *p* = 0.189; quadratic, *p* = 0.208) indicating that CE remained relatively stable during this timeframe.

#### MEP amplitude during the 3^rd^ epoch

Figure 2 demonstrates a substantial increase in CE following the IS as the movement was executed. In order to characterize this increase, a one-way, six factor (Time: 0 ms, +25 ms, +50 ms, +75 ms, +100 ms, +125 ms) repeated measures ANOVA was conducted to examine any differences in MEP amplitude occurring following the IS. A significant main effect of time was found, (*F*(5,85) = 9.783, *p*<0.001, η_p_^2^ = 0.365), as well as a significant linear trend (*F*(1,17) = 12.887, *p* = 0.002, η_p_^2^ = 0.431) and a significant quadratic trend (*F*(1,17) = 19.574, *p*<0.001, η_p_^2^ = 0.535). Post-hoc analyses using Bonferonni corrected student's t-tests were used to determine the locus of the differences. The results indicated a significant difference between time point 2 (+25 ms) and time points 5 (+100 ms) and 6 (+125 ms), time point 3 (+50 ms) and time points 6 (+125 ms), and time points 4 (+75 ms) and time point 6 (+125 ms), confirming a significant increase in CE following the presentation of the IS (figure 5).

#### MEP amplitude relative to the onset of EMG

A final data analysis investigated the MEP amplitude data time-locked to the onset of EMG as RTs varied from trial to trial and among participants. For this analysis, similar data reduction techniques from prior studies [12] were used. Specifically, MEP amplitudes were expressed as a percentage of each participant's maximal MEP amplitude, and organized by onset time into 7 time bins relative to EMG onset by calculating the time difference between the onset of EMG and the MEP. These time bins included MEP onsets that occurred either <60 ms prior to EMG onset or 10 ms time bins at increasing intervals up to 110 ms prior to EMG onset (see figure 6). Nine participants were missing data points for the <60 ms bin and were thus excluded from this analysis, resulting in a data set including 9 of the original 18 participants. Because one participant did not have a value for the 100–110 ms time bin, this missing value (1 out of 63) was filled using a linear-regression based multiple imputations procedure in SPSS (IBM Inc.). A one-way repeated measures ANOVA was conducted between 7 time bins (<60 ms, 60–70 ms, 70–80 ms, 80–90 ms, 90–100 ms, 100–110 ms, and >110 ms) relative to the onset of EMG. The results indicated a significant main effect of time (*F*(6,48) = 3.485, *p* = 0.006, η_p_^2^ = 0.303). Post-hoc analyses using Bonferonni corrected student's t-tests indicated significant differences between the <60 ms time bin and both the 70–80 ms and 80–90 ms time bin, indicating that CE began to increase approximately 70 ms prior to EMG onset, in accordance with previous studies. These results are shown in figure 6.

## Discussion

**Figure 4:**
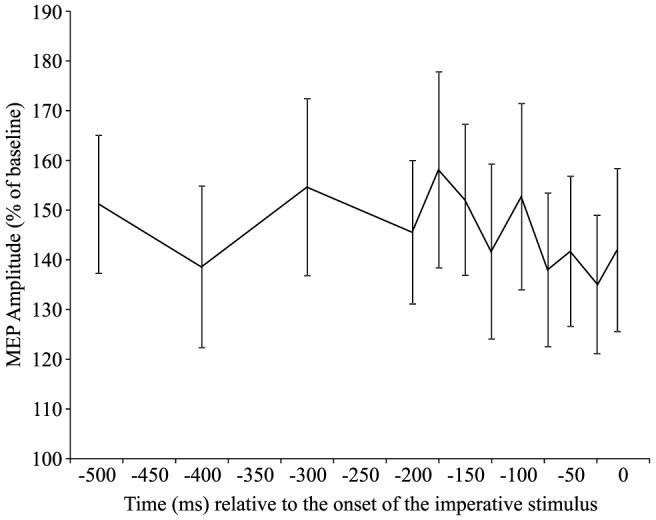
Mean (+/− SE) motor-evoked potential (MEP) amplitude expressed as a percentage of baseline for the time encompassing −500 ms to 0 ms relative to the onset of the imperative stimulus. No significant differences in MEP amplitude were found during this time period.

**Figure 5:**
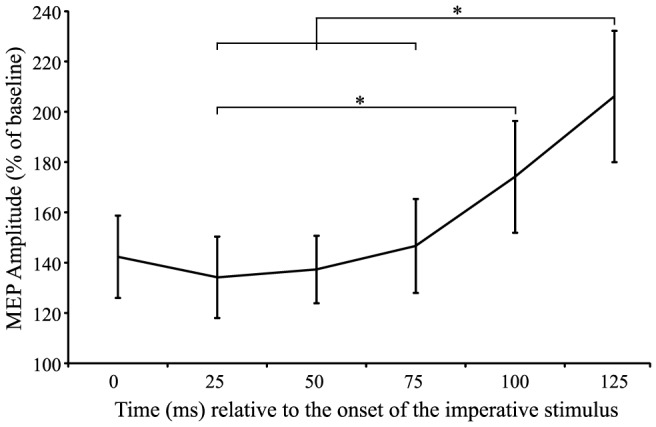
Mean (+/− SE) motor-evoked potential (MEP) amplitude expressed as a percentage of baseline for the time encompassing 0 ms to +125 ms relative to the onset of the imperative stimulus. Asterisks (*) denote significant differences in MEP amplitude during the initiation phase.

**Figure 6:**
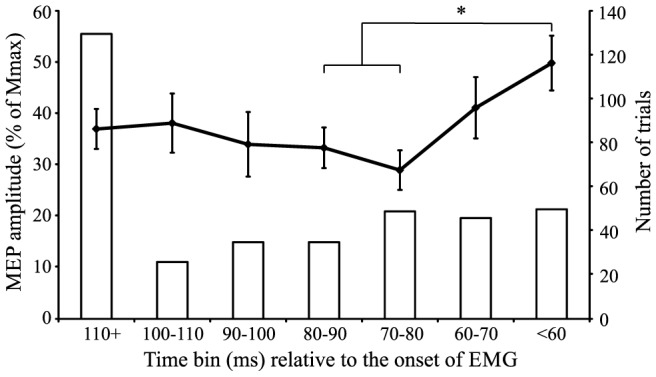
Mean (+/− SE) motor-evoked potential (MEP) amplitude during the RT interval expressed as a percentage of maximal MEP (Mmax) for 10 ms time bins prior to EMG onset. The number of trials making up the mean for each of the time points (secondary axis) is represented by columns. The asterisk (*) denotes a significant increase in MEP amplitude as EMG onset approaches.

The purpose of this study was to examine the time course of corticospinal excitability (CE) across the combined preparatory and initiation phases of movement execution in a simple RT task. Previous work has only examined CE independently during the foreperiod (prior to the IS) and the reaction-time interval (following the IS), which does not allow for a direct comparison between these two time periods. In addition, past studies have described changes in CE in relation to previous time points, without comparing these to a “true” resting baseline level. The results of the current study indicate that in a simple RT task with a moderately long fixed foreperiod, CE increases from a resting level within the first 500 ms following the WS, which is then held at a relatively consistent level until the presentation of the IS (figs. 3 & 4). After presentation of the IS, and as the participant initiates the response, excitability increases dramatically from this elevated state (figs. 5 and 6). The relatively short premotor RT (185 ms±21 ms) indicates that participants were preparing for the upcoming movement and initiating the response quickly without anticipatory false starts, thus allowing for an accurate description of CE associated with the preparatory and initiation processes.

Models of neural activation describing motor preparation have suggested that cortical excitability increases from a baseline level approximately 1 s prior to the anticipated go-signal and is held below threshold until the go-signal is received [8], [22], [23]. Motor preparation has been previously envisioned as the increase in activity of a neural network, or “cell assembly,” up to an initial steady state which is held at a level below threshold (i.e. the “ignition” point) [3]. Consistent with these models, the current study found that during the foreperiod there was an increase in MEP amplitude to approximately 160% of inter-trial baseline values, and that this heightened state was maintained until the IS (figs. 3 & 4). Although previous studies have used TMS to examine excitability in the foreperiod of RT tasks, the current study compared excitability throughout the trial to CE measured during a long and variable inter-trial interval. In contrast, previous studies have examined the level of excitability in comparison to various different control conditions, in which it is possible (and even likely) that preparatory activation had already begun. For example Touge and colleagues [13] compared CE measured during the foreperiod to that measured in trials where no warning signal was given; however, due to the experimental design it is likely that participants were in a state of perpetual heightened readiness. Thus, the current data provides unique insight into the time course of CE changes underlying motor preparation during the foreperiod, with respect to a resting baseline level.

Previous work has shown a decrease in MEP amplitude during the foreperiod [13], [21], and a visual inspection of Figure 4 shows a similar effect in the 500 ms preceding the IS, although neither the effect of time (*p* = 0.434) or trend analyses (linear, *p* = 0.189; quadratic, *p* = 0.208) were statistically significant. However, the present study employed a 1500 ms foreperiod, which is substantially longer than that used in some earlier studies (e.g., 500 ms, [13], [21]). It has been suggested that the onset of response preparation and the temporal dynamics of the excitability curve may depend the temporal predictability of the response time [22], [24], with a longer foreperiod making it more difficult to anticipate the upcoming IS [25]. The decrease in CE shown previously when shorter foreperiods were used may reflect inhibitory processes that are engaged to prevent a premature ignition of the cell assembly, and thus early release of the action (i.e. false starts). While “inhibition” is a rather large concept, Aron's [26] notion of selective inhibition might best describe the processes occurring in previous studies of foreperiod CE, as it implies a mechanism that allows for the suppression of specific actions. More specifically, a proactive selective inhibitory control implies the preparation to stop a specific, upcoming response. In contrast, and consistent with the current study, CE has been shown to remain much more stable in RT tasks that involve a longer foreperiod [13] as the increased temporal uncertainly of the IS may not require the same degree of suppressive inhibitory activity, thus resulting in a less robust decrease in CE.

Activation models describing motor preparation and initiation, such as the ones noted above [8], [23], typically include 3 phases: an initial increase, a maintenance phase prior to the IS, and a final increase in conjunction with the presentation of the IS resulting in release of the movement. While it has been shown that the MEP data observed in the current study mirrors the initial preparation and hold phases of an activation model, it also depicts a final increase in activation following the IS (fig. 5). This increase in activation can be described both as linear (*p* = 0.002) and quadratic (*p*<0.001) in nature, although visual inspection of Figure 5 suggests that a quadratic, or accelerating rate of increase in CE, best describes the data. Importantly, as CE was examined throughout the entirety of the time course leading up to movement execution the current study is able to provide context to the final phase of the movement initiation. Previous studies often describe CE in the RT interval (between the go-signal and EMG onset) either in terms of a percentage of maximal MEP amplitude [12], or as a percentage of CE measured at the go-signal. However, the present data shows a similar increase in CE following the IS, but this is from a heightened state (130–140%) with respect to rest (fig. 2). In addition, this aspect of excitability is often obscured as MEPs are often expressed as time-locked to EMG onset. This is for good reason, since RTs can be variable, leading to variable amounts of excitability at a similar time following the IS. For this reason, the present data were also expressed with respect to when the MEP occurred with respect to EMG onset (fig. 6). In this analysis the MEPs measured during the RT interval also closely follow those of previous studies [9]–[12] in which MEP amplitude increases roughly 60–80 ms prior to the onset of EMG.

In summary, the current study was designed to provide a more complete description of CE changes that occur during a simple RT task, beginning from resting values until the start of movement execution. Overall, the data indicates that CE quickly increases from baseline values, coinciding with the presentation of the WS, indicative of preparatory processes. This is followed by a holding period in which CE is maintained at a relatively consistent heightened level prior to the presentation of the IS, and by a rapid increase in CE from this elevated level as the movement is initiated and released. These results are in line with previous studies [13], [21] that have shown that CE remains stable prior to the presentation of the IS in RT tasks involving an unpredictable foreperiod, as well as previous studies [9]–[12] that have shown an increase in CE as EMG onset approaches, following the IS. However, this study provides a much more cohesive picture of CE during a RT task, and the described excitability time course is also consistent with neural activation models that describe movement preparation and response initiation processes [5]–[8].
